# A weak post‐acidification *Lactobacillus helveticus* UV mutant with improved textural properties

**DOI:** 10.1002/fsn3.2016

**Published:** 2020-11-15

**Authors:** Chengran Guan, Xuan Chen, Ruifeng Zhao, Yuan Yuan, Xinyuan Huang, Jianbo Su, Xiangli Ding, Xia Chen, Yujun Huang, Ruixia Gu

**Affiliations:** ^1^ Key Lab of Dairy Biotechnology and Safety Control College of Food Science and Technology Yangzhou University Yangzhou China

**Keywords:** fermented milk, *L. helveticus*, postacidification, texture, UV mutation

## Abstract

To derive a mutant of *L. helveticus* SH2‐1 with the capacity of weak postacidification and high texturing, first, taking *L. delbrueckii* frs4‐1 and *S. thermophilus* grx02 as the controls, H^+^‐ATPase activity was demonstrated to be highly related to the postacidification of *L. helveticus* SH2‐1. Then, by detecting H^+^‐ATPase activity, the weak postacidify mutant of *L. helveticus* SH2‐1 (renamed as *L. helveticus* sh2‐5–66) was selected from 80 UV mutants. The pH and acidity of the milk fermented with *L. helveticus* sh2‐5–66 were separately 0.57 pH units higher and 57.1 °T lower than that of *L. helveticus* SH2‐1. The acidification of *L. helveticus* sh2‐5–66 was further demonstrated to be genetically stable during 100 generations cultivation. Moreover, the milk fermented with *L. helveticus* sh2‐5–66 showed improvement in textural and rheological properties and flavor during storage which could be further improved by coculture with the commercial starter *S. thermophilus* st447.

## Graphical abstract

H+‐ATPase activity was demonstrated to be highly related to the postacidification of *L. helveticus* SH2‐1. By detecting H+‐ATPase activity, the weak postacidify mutant renamed as *L. helveticus* sh2‐5–66 was selected from 80 UV mutants. The milk fermented with *L. helveticus* sh2‐5–66 showed improvement in textural and rheological properties and flavor during storage.

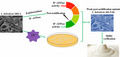

## 1 INTRODUCTION

Fermented dairy products are becoming increasingly popular foods because of their high nutritional value, unique flavor, desirable benefits to human health and their specific sensory and texture characteristics (Esmerino et al., 2017; Koskinen et al., 2018). Fresh fermented products are generally stored at temperatures between 4°C and 8°C for a period of 4 weeks. Although some metabolic activity of the fermented bacteria is reduced by cold storage, the remaining activity leads to the production of lactic acid and results in a decrease in pH and an increase in acidic flavor (the process of postacidification) (Donkor et al., 2006). Postacidification will shorten the shelf life and cause several adverse effects on the quality of the products, such as a strong acidic taste, increased whey separation, and decreased lactic acid bacteria count (Settachaimongkon et al., 2016; Zhang et al., 2012). It is a major problem in manufacturing and during storage prior to consumption.

Reported studies on controlling postacidification can be classified as controlling bacterial growth by physical and chemical methods and changing the capacity of acid production by genetic engineering or random mutagenesis. To control growth, the bacteria could be killed after fermentation by physical methods such as pasteurization and ultrahigh pressure, or the activity of survivors of starter cultures could be restrained during the storage period by the addition of preservatives such as potassium sorbate and bacteriocins (Beal et al., 1999; Han et al., 2012; Liu et al., 2013). Regarding genetic methods, genetic engineering has been used to change or modify the route of acid production, such as altering cell membrane permeability (Dan et al., 2015; Wang et al., 2013). However, the bacteria produced by genetic engineering are not allowed to be used in food production in some countries. In contrast, bacteria obtained by random mutagenesis could be acceptable.

Mutagenesis for strains with weak postacidification mainly focus on the species of *Lactobacillus delbrueckii* subsp. *bulgaricus* and *Streptococcus thermophilus,* which are essential culture components in the production of fermented dairy products, especially yogurt. Mutant strains of *L. delbrueckii* subsp. *bulgaricus* combining excellent texturizing properties with low postacidification have been reported to be generated using UV light or chemical mutagens such as ethane methane sulfonate (EMS) and N‐methyl‐N'‐nitro‐N‐nitroguanidine (NTG). Isolated mutants of *L. delbrueckii* subsp. *bulgaricus* treated with diethyl sulfate and ultraviolet light had 5.9% and 11.9% lower postacidification capacities, respectively, than the original strain at 25°C (Zhang et al., 2012). Additionally, weak postacidification *S. thermophilus* variants deficient for oligopeptide transport were selected using a toxic peptide analog. The variants have rapid acidification kinetics allowing a high degree of acidity and do not postacidify during the course of fresh fermented dairy storage (Garault et al., 2006). In addition, Margolles and Sanchez (2012) characterized a new strain, *Bifidobacterium animalis* subsp. *lactis* CECT 7,953, obtained by random UV mutagenesis, which produces less acetic acid than the wild‐type strain (Margolles & Sanchez, 2012). In addition to all the above results, there are hardly any genetic mutagenesis studies on controlling the postacidification of fermented strains in other species.

*Lactobacillus helveticus* is a homofermentative, thermophilic lactic acid bacterium widely used in the manufacture of Swiss‐type and Italian aged cheeses and fermented milk drinks (Gatti et al., 2004; Vinderola et al., 2007). *Lactobacillus helveticus* has the ability to reduce bitterness and give a characteristic flavor to cheese, which makes this bacterium an important component of starter cultures for the dairy industry. In our lab, *L. helveticus* SH2‐1 was isolated and found to be a prominent probiotic. However, *L. helveticus* SH2‐1 was significantly postacidifying for dairy products. In this work, a selection method was first established to isolate weak postacidifying mutants after UV mutagenesis of *L. helveticus* SH2‐1. Then, the potential of the mutant applied to dairy products was inspected. The aim of this study was to isolate the *L. helveticus* SH2‐1 mutant that not only reduced postacidification but also contributed to an improved texture of dairy products.

## 2 MATERIALS AND METHODS

### 2.1 Strains and growth conditions

*Lactobacillus helveticus* SH2‐1, *L. delbrueckii* frs4‐1, and *S. thermophilus* grx02 were persevered in the laboratory, and *S. thermophilus* st447 was purchased (Haoyue Co. Ltd.). The Lactobacillus and Streptococcus strains were separately subcultured through two transfers in MRS and M17 medium at 42°C for 12 hr and stored at 4°C. One milliliter of prepared microbial suspension of the last transfer was inoculated into 10 ml of appropriate medium and was used to screen the mutant. To prepare the milk fermentation, 1 ml of the subcultivated strain was inoculated in tubes containing 9 ml of sterilized reconstituted skim milk powder (12%, w/v). The inoculated tubes were mixed in a vortex and incubated at 42°C for coagulation.

### 2.2 Detection of cell growth, acidity, and pH

The detection of viable bacterial counts was carried out as described with slight modification (Oliveira et al., 2009). One milliliter of the sample was diluted with 9 ml of saline solution, and eight serial dilutions were performed. Each bacterium was counted in the three most appropriate dilutions by applying the pour plate technique (Kodaka et al., 2005). Counts are presented as the mean values. Streptococcus and Lactobacillus colonies were counted after aerobic incubation at 37°C for 48 hr. The acidity was determined by the volume of 0.10 mol/L NaOH solution required to titrate the sample to neutral pH. The pH was measured using a laboratory pH meter (Mettler Toledo FE20). Each trial was performed in triplicate.

### 2.3 Measurement of enzymatic activity

Fermented milk (10.0 g) was blended well with 15 ml of EDTA solution (1% w/v, pH 12) and then centrifuged at 10,000 rpm for 5 min to obtain the cells. After washing three times with phosphate buffer (20 mmol/L, pH 7.0), the cells were resuspended in 10 ml of TE buffer (20 mmol/L, pH 7.0) and processed for 1 hr at 37°C after the addition of lysozyme (0.6 ml, 0.2 mmol/L). Then, the cells were recovered by centrifugation and resuspended in saline solution with the same OD_600_. Disruption of the cells was performed with an ultrasonic homogenizer (scientz‐IID, Ningbo, China). The disruption period was 5 s in 3 s intervals for a duration of 1 min, and the samples were kept in an ice bath during cell disruption to prevent overheating. H^+^‐ATPase activity was measured as described by the H^+^‐ATPase assay kit (JianCheng Bioengineering Institute). β‐Galactosidase activity was assayed as previously described (Guan et al., 2020).

### 2.4 Random mutagenesis of *L. helveticus* SH2‐1 and screening of the mutant

*Lactobacillus helveticus* SH2‐1 was cultivated to the logarithmic growth phase, and the cells were isolated by centrifugation (10,000 ×*g*, 10 min). The cells were washed twice with 0.85% sterilized saline solution and then diluted to prepare the cell suspension (1 × 10^8^ CFU/ml). Five milliliters of the suspension was added to a petri dish with a diameter of 9 cm. The dish was put 30 centimeters directly underneath a 20‐watt UV light, which had been preheated for 30 min. After exposure for 0 s, 30 s, 60 s, 90 s or 120 s, the cells were isolated by centrifugation for 10 min (4°C, 10,000 ×*g*). Then, the cells were suspended in 5 ml of MRS medium for cultivation in the dark for 12 hr at 42°C, and viable counts were detected on solid MRS medium. Using the viable counts of the sample exposed for 0 s (N_0_) as the control, the lethality rate (S) of the sample exposed for different times (N_t_) was calculated as follows: S= (N_0_‐N_t_)/N_0_ × 100%.

Dishes with a lethality rate higher than 80% but lower than 90% were selected to isolate the mutant. Fifty‐five colonies were chosen from these dishes and separately incubated in MRS medium and cultivated at 42°C for 48 hr. Strains were harvested by centrifugation at 10,000 ×*g* for 5 min, washed three times with saline solution and suspended in double distilled water. Disruption of the strains and detection of the H^+^‐ATPase activity of each sample were performed as described above.

### 2.5 Genetic stability of the mutant

The selected mutant was incubated for 12 hr in MRS medium. Then, 6% of the culture was transferred to new MRS medium and cultivated for 8 hr (defined as the first generation). The mutant was continuously subcultivated for 100 generations according to the above method. Compared with the mutant, the 5th, 10th, 15th, 20th, 40th, 60th, and 100th subcultivated strains were inoculated into MRS medium and tested for their pH value per the usual procedure.

### 2.6 Texture analysis

The texture of the fermented milk was assessed as described with some modifications (Kumar & Mishra, 2003). Eighty milliliters of fermented milk samples contained in a 100 ml plastic container was used to assess yogurt texture using a TMS‐Pro texture analyzer (Food Technology Corporation) equipped with a 12.7 mm diameter cylindrical probe. Samples were kept in an ice box until their measurement at room temperature. The contact area was set at 1 mm^2^, and the contact force was set to 0.05 N. The instrument speed was set at 1 mm/s. The compression distance, which is the distance of penetration from the surface of the sample, was set at 15 mm. The hardness, cohesiveness, springiness, adhesiveness, gumminess, and chewiness of the fermented milk were determined by the maximum force measured during sample compression.

Viscosity. All samples of the fermented milk were stirred with a glass bar 10 times clockwise and 10 times counterclockwise prior to measurement. The viscosity was detected as described by the operation instructions of a viscometer (RVDV‐II+, Brookfield, Engineering laboratories, Inc.) allocated with rotor 4 and a rotation speed of 35 rpm.

Water holding capacity (WHC). Ten grams of fermented milk was centrifuged (3,500 rpm, 15 min) in a preweighed tube (mt). The water was discarded, and then the tube was turned upside down for 10 min to further remove the remaining water. After that, the tube was weighed (ms), and the WHC was calculated by the following equation:

$$\mathrm{WHC}\left(\%\right)=\frac{\mathrm{ms}}{\mathrm{mt}+10}\times 100$$

Rheology. Using a Kinexus pro rotational rheometer (Malvern Instruments Ltd.), the rheological properties of yogurt during storage were determined according to a method described in a previous study with slight modifications (Purwandari & Vasiljevic, 2009). All samples were stirred with a glass bar 10 times clockwise and 10 times counterclockwise prior to loading to achieve a homogenous mixture. The shear rate increased logarithmically from 0.1 to 100/s in 120 s (upward curve) followed by a logarithmic decrease from 100 to 0.1/s in 120 s (downward curve). Data were collected every 2 s. The thixotropy (hysteresis loop area) between the upward and downward flow curves was determined.

### 2.7 Flavor substance measurement by head space chromatography

Four milliliters of fermented milk samples were collected for centrifugation (6,000 rpm, 15 min). One milliliter of the supernatant was collected and filtered through a 0.22 µm microporous membrane. The flavor substance acetaldehyde and diacetyl content in the sample were determined by the HS‐GC method with some modifications (Tian et al., 2017). A GC system (Agilent 7,890) coupled to an MS detector (Agilent 7697A) and silica capillary column (30 m × 0.25 mm × 0.25 μm) was used for the separation and detection of the flavor compounds. The column temperature was maintained at 40°C for 2 min and then increased to 100°C at a rate of 3°C/min and then further increased to 200°C at a rate of 30°C/min. The oven temperature program started at 40°C for 2 min and was followed by a 3°C/min increase to 50°C and then a 6°C/min increase to 100°C, which was maintained for 5 min. The injector temperature was set to 160°C, and the flame detector was set to 250°C. Nitrogen was used as the carrier gas with a constant flow rate of 5 ml/min. The concentrations of the flavor volatiles analyzed in the fermented substrates were determined by comparison with external calibration curves of acetaldehyde and diacetyl.

### 2.8 Confocal laser scanning microscopy analysis

The microstructure of the fermented milk was detected using a confocal laser scanning inverted microscope (LSM 880NLO, Zeiss) with a He/Ne 5 Laser and 63× (oil) objective. Sample preparation followed the method described by Kristo et al (Kristo et al., 2011). The fermented milk was homogeneously stirred, and then 2 ml of the milk was mixed with ten microliters of a 0.2% (w/v) aqueous solution of rhodamine B to stain the protein network. A few drops of the mixture were transferred to a slide with a cavity and covered with a cover slip. The slide carrying the stained mixture was placed in an incubator at 37°C until the sample pH reached 4.6. The slide was stored overnight at 4°C before observation by CLSM.

### 2.9 Data analysis

All the experiments were repeated three times unless stated otherwise. Statistical analysis for the results was done using Student's *t* test (*p* < .05).

## 3 RESULTS AND DISCUSSION

### 3.1 Preparation of the mutant of *L. helveticus* SH2‐1 with weak postacidification properties

#### 3.1.1 Development of a method for the isolation of a weak postacidification mutant strain

**FIGURE 1:**
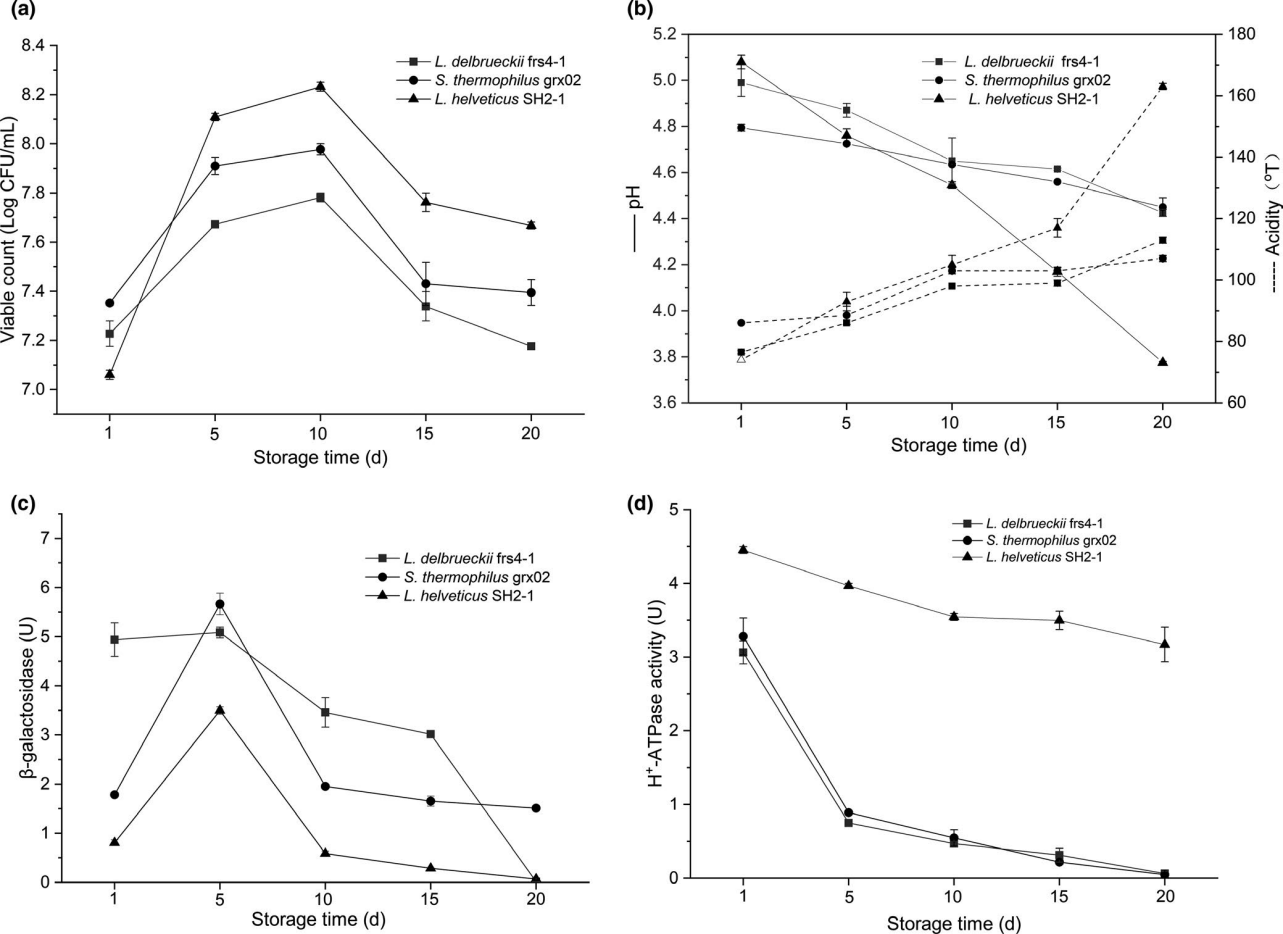
Growth curve (a), pH and acidity (b), β‐galactosidase activity (c) and H^+^‐ATPase activity (d) of*L. delbrueckii*frs4‐1,*S. thermophilus*grx02 and*L. helveticus*SH2‐1

Single fermented milks with *L. delbrueckii* frs4‐1, *S. thermophilus* grx02 and *L. helveticus* SH2‐1 were separately prepared. The viable counts, pH and acidity of the corresponding fermented milks during storage for 20 days were measured (Figure 1a,b). These three strains exhibited similar variation trends in growth and acidification. The viable counts continued to increase during storage for 10 days but then decreased, and the pH and acidity of the fermented milk continued to decrease and increase, respectively, during the whole storage period. The acidity and pH of the milk fermented by *L. delbrueckii* frs4‐1 and *S. thermophilus* grx02 were lower than 120°T and higher than 4.42, respectively, during the storage period. However, the pH and acidity of *L. helveticus* SH2‐1 significantly changed after storage for 10 days, and the pH was lower than 3.77, and the acidity was higher than 163 °T after storage for 20 days. Therefore, *L. delbrueckii* frs4‐1 and *S. thermophilus* grx02 could be used as controls to identify the enzyme highly related to the acidification of *L. helveticus* SH2‐1.

Acid production involves a large enzymatic system, among which β‐galactosidase metabolizes lactose into lactic acid and H^+^‐ATPase catalyzes the movement of hydrogen ions across the cell membrane, playing an important role in postacidification (Iskandar et al., 2019; Wang et al., 2013). Therefore, to select the enzyme that was highly related to the postacidification of *L. helveticus* SH2‐1, the enzymatic activities of β‐galactosidase (Figure 1c) and H^+^‐ATPase (Figure 1d) were tested during the storage period. There was no large difference between *L. helveticus* SH2‐1 and control strains *L. delbrueckii* frs4‐1 and *S. thermophilus* grx02 in the β‐galactosidase activity assay. However, in the H^+^‐ATPase activity assay with *L. helveticus*, the H^+^‐ATPase activity decreased slowly during storage and was significantly higher than that of the two control strains, which continued to decrease quickly and was hardly detected after 5 days of storage. The difference in the H^+^‐ATPase activity between *L. helveticus* SH2‐1 and control strains *L. delbrueckii* frs4‐1 and *S. thermophilus* grx02 was closely related to the profile of the postacidification of these three strains. Therefore, H^+^‐ATPase activity was used to select the weak postacidification mutant of *L. helveticus* SH2‐1 in the next experiments.

### 3.2 UV mutation and isolation of the weak acidification mutant

Traditional mutation methods, such as ultraviolet (UV) light or chemical mutagens, are widely used in genetic mutagenesis. UV mutagenesis is preferred because it is easy to control and no toxic waste is produced. It is also a relatively mild mutagenesis, so the risk of undesirable second site mutations is lower than that with chemical mutagenesis. In this study, UV mutagenesis was used to obtain a mutant of *L. helveticus* SH2‐1 with weak acidification properties. First, the UV exposure time and the lethality rate were measured (Table S1). As the lethality rates of *L. helveticus* SH2‐1 exposed for 50 s and 60 s were 87% and 99%, respectively, 55 colonies were selected from these two samples. After cultivation in MRS for 48 hr, the H^+^‐ATPase activity of these colonies was measured, and the enzyme activity ranged from 0.03 U to 5.25 U (Table S2). Furthermore, 20 colonies with H^+^‐ATPase activity lower than that of the wild‐type strain (*L. helveticus* SH2‐1, 2.32 U) were used to prepare fermented milk. Only 10 colonies coagulated, and the corresponding fermented milks exhibited weaker acidification than the wild strain (Table 1). The results suggested that the detection of H^+^‐ATPase activity is an effective and cost‐saving method to isolate weakly acidifying mutants of *L. helveticus* SH2‐1 after UV mutagenesis.

**TABLE 1:** The postacidification of the mutant strains

Colony	Acidity				pH			
	1 day	3 days	5 days	20 days	1 day	3 days	5 days	20 days
C^1^	74 ± 0.1	82.0 ± 2.0	93.0 ± 3.0	162.5 ± 0.5	5.08 ± 0.03	4.85 ± 0.02	4.68 ± 0.03	3.73 ± 0.04
8	71 ± 0.1	76.5 ± 0.5	87.7 ± 0.1	121.0 ± 0.8	5.10 ± 0.02	4.97 ± 0.01	4.71 ± 0.01	3.99 ± 0.01
10	70.5 ± 0.5	77.0 ± 3.0	86.7 ± 1.1	120.4 ± 0.2	5.10 ± 0.01	4.98 ± 0.02	4.83 ± 0.01	4.11 ± 0.01
12	68.5 ± 0.5	74.7 ± 0.3	82.5 ± 0.5	114.4 ± 0.6	5.11 ± 0.01	4.97 ± 0.02	4.83 ± 0.01	4.16 ± 0.01
13	70.5 ± 0.5	76.6 ± 0.2	86.4 ± 1.4	122.0 ± 0.4	5.10 ± 0.01	4.99 ± 0.02	4.74 ± 0.03	4.1 ± 0.01
24	71.5 ± 0.5	81.5 ± 0.5	91.0 ± 1.0	123.2 ± 0.2	5.03 ± 0.03	4.91 ± 0.02	4.61 ± 0.01	3.98 ± 0.03
26	69.1 ± 1.1	76.7 ± 0.5	87.1 ± 4.1	121.8 ± 1.2	5.11 ± 0.01	4.97 ± 0.02	4.82 ± 0.02	4.12 ± 0.02
27	70.0 ± 1.0	76.8 ± 4.4	88.6 ± 0.1	122.5 ± 1.4	5.01 ± 0.02	4.94 ± 0.03	4.78 ± 0.02	4.09 ± 0.02
41	69.9 ± 0.3	76.5 ± 0.5	87.2 ± 0.2	120.3 ± 0.5	5.01 ± 0.01	4.96 ± 0.03	4.70 ± 0.02	4.12 ± 0.03
42	71.6 ± 0.2	79.5 ± 0.5	90.3 ± 0.7	122.5 ± 1.1	5.08 ± 0.01	4.96 ± 0.02	4.72 ± 0.02	4.01 ± 0.03
52	65 ± 0.5	72.5 ± 0.6	80.6 ± 0.1	105.4 ± 0.5	5.15 ± 0.03	5.03 ± 0.02	4.82 ± 0.03	4.30 ± 0.02

Note

C^1^: The wild‐type *L. helveticus* SH2‐1.

In particular, mutant 52 was selected, which showed the lowest H^+^‐ATPase activity, and its corresponding fermented milk had the highest pH value and lowest acidity among all the mutants. The pH and acidity of the milk fermented with mutant 52 stored for 20 days were 4.3 and 105.4 °T, respectively, which were 0.57 pH units higher and 57.1 °T lower than that of *L. helveticus* SH2‐1. The reported weak postacidification patent strain CHCC8535 showed a pH of 4.17 after 20 hr, which was 0.32 pH units higher than that of the wild‐type strain, and CHCC7159 showed a pH of 4.64 after 20 hr, which was 0.50 pH units higher than that of the wild‐type strain (Jensen & AH, 2007). Compared with these wild‐type strains, mutant 52 showed great potential for weak postacidification fermented products. After sequence blasting of the 16S rDNA, mutant 52 was renamed *L. helveticus* sh2‐5–66 and stored in the China General Microbiological Culture Collection Center.

### 3.3 Acid‐producing stability of *L. helveticus* sh2‐5–66

**FIGURE 2:**
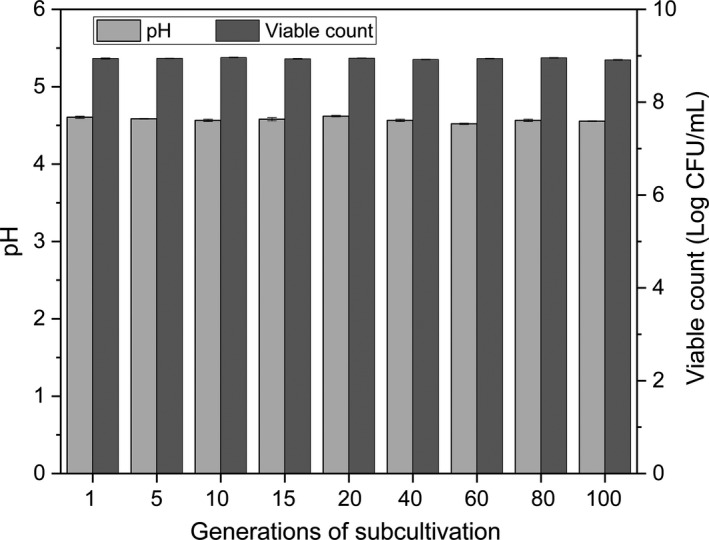
pH and viable counts of the generations of*L. helveticus*sh2‐5–66 during continuous passage culture

Genetic stability of the mutants is essential for the fermentation industry and reflects the occurrence of mutations at the gene level (Saarela et al., 2011). The usual method used for detection of the mutant's stability is to test the required parameters of the specific generations during continuous passage cultures (Wang et al., 2010). Therefore, *L. helveticus* sh2‐5–66 was processed in a 100‐generation culture, and the 5th, 10th, 15th, 20th, 40th, 60th, 80th and 100th generations were separately processed for the detection of acidification stability (Figure 2). There were few differences in pH (0.1 pH unit) and viable counts (0.05 Log CFU/ml) between the subcultured samples and *L. helveticus* sh2‐5–66. The results indicated that *L. helveticus* sh2‐5–66 was genetically stable.

### 3.4 Performance of *L. helveticus* sh2‐5–66 used for fermented milk

A trend in the starter market for fermented milks is a starter combining a low postacidification profile and a high texturing profile during the shelf life. To test the industrial potential of *L. helveticus* sh2‐5–66 (abbreviated as sh2‐5–66), single and coculture fermentations were performed. For the coculture fermentation, *S. thermophilus* st447 (abbreviated as st447) was used. The wild‐type strain *L. helveticus* SH2‐1 (abbreviated as SH2‐1) was used as the control.

For preparation of the single fermented milk, strains SH2‐1, sh2‐5–66 and st447 were separately processed according to the usual methods. During preparation of the cofermented milks, SH2‐1 and sh2‐5–66 were separately mixed with st447 at the optimized ratio of 2:1 (the data for the blending ratio optimization are shown in Figure S1). In addition to acidity, the texture characteristics, rheological properties and sensory flavor of the fermented milks are critical parameters in sensory evaluation and consumer acceptability. Therefore, these characteristics of the fermented milks after 20 days of storage were detected.

### 3.5 Viable counts, pH and acidity of the fermented milks

**FIGURE 3:**
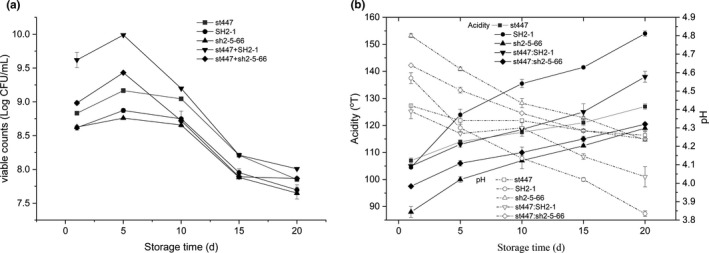
Viable counts (a) and pH and acidity (b) of the fermented milks with different strains

The viable counts, pH and acidity of the fermented milks were measured (Figure 3). There were differences at the beginning of storage (day 1), while the variation trends were similar during the storage period (from day 1 to day 20). The strains in each sample continued to grow before day 5 and then decreased. In this study, the viable counts on day 20 were higher than 7.5 Log CFU/ml. Moreover, the pH and acidity of the samples continued to decrease and increase during storage, respectively. Combining the pH and acidity, the acidification profile of the pure and cofermented milk of strain sh2‐5–66 was significantly improved compared with the milk fermented with strain SH2‐1. The pH of the pure and cofermented milk of strain sh2‐5–66 was 4.26 and 4.24, respectively, after 20 days of storage, and the corresponding variation in ∆pH during 20 days of storage was 0.57 and 0.4, respectively. These results were similar to the reported weak acidification parent strain CHCC7159, which had a pH in the range of 4.25–4.55 after 14 days and/or after 21 days (Jensen & AH, 2007).

### 3.6 Texture profile of the fermented milks

The textural properties of the fermented milks were analyzed by texture profile analysis (TPA), including the hardness, cohesiveness, springiness, gumminess, and chewiness (Table 2). These indexes of the five samples almost all increased before 5 days and then decreased. According to these five texture indexes of the fermented milk, st447 showed the best performance, while sh2‐5–66 performed slightly better than sh2‐5. The texture of the single fermented milk with sh2‐5–66 and SH2‐1 could be further improved by coculture with st447.

**TABLE 2:** Texture characteristics of fermented milks during storage phase

Index	Storage period	Bacteria				
		st447	SH2−1	sh2−5–66	st447: SH2−1	st447: sh2−5–66
Hardness/*N*	1 day	0.18 ± 0.002	0.12 ± 0.005	0.14 ± 0.009	0.15 ± 0.004	0.15 ± 0.002
	5 days	0.32 ± 0.006	0.15 ± 0.008	0.18 ± 0.001	0.17 ± 0.006	0.21 ± 0.002
	10 days	0.28 ± 0.006	0.13 ± 0.00	0.16 ± 0.008	0.16 ± 0.003	0.18 ± 0.004
	15 days	0.26 ± 0.003	0.13 ± 0.002	0.14 ± 0.004	0.16 ± 0.01	0.17 ± 0.006
	20 days	0.25 ± 0.004	0.11 ± 0.006	0.13 ± 0.006	0.15 ± 0.004	0.16 ± 0.001
Cohesion/Ratio	1 day	0.53 ± 0.02	0.47 ± 0.01	0.48 ± 0.01	0.45 ± 0.01	0.48 ± 0.01
	5 days	0.58 ± 0.01	0.54 ± 0.02	0.55 ± 0.02	0.53 ± 0.03	0.53 ± 0.01
	10 days	0.55 ± 0.02	0.49 ± 0.01	0.54 ± 0.02	0.48 ± 0.01	0.50 ± 0.01
	15 days	0.5 ± 0.01	0.47 ± 0.01	0.49 ± 0.01	0.48 ± 0.02	0.50 ± 0.01
	20 days	0.47 ± 0.02	0.44 ± 0.01	0.48 ± 0.02	0.47 ± 0.01	0.50 ± 0.01
Springiness/mm	1 day	7.30 ± 0.08	7.40 ± 0.13	8.80 ± 0.07	7.86 ± 0.05	8.99 ± 0.37
	5 days	9.31 ± 0.30	8.46 ± 0.12	11.31 ± 0.63	12.85 ± 0.51	12.62 ± 0.26
	10 days	13.65 ± 0.79	7.65 ± 0.21	7.66 ± 0.07	10.16 ± 0.42	10.22 ± 0.83
	15 days	8.69 ± 0.34	7.39 ± 0.17	7.54 ± 0.43	8.75 ± 0.13	9.02 ± 0.28
	20 days	8.52 ± 0.01	5.94 ± 0.23	6.98 ± 0.12	8.31 ± 0.19	8.77 ± 0.02
Gumminess/*N*	1 day	0.09 ± 0.003	0.055 ± 0.003	0.069 ± 0.003	0.067 ± 0.002	0.072 ± 0.001
	5 days	0.183 ± 0.005	0.078 ± 0.006	0.099 ± 0.002	0.091 ± 0.007	0.11 ± 0.001
	10 days	0.098 ± 0.006	0.065 ± 0.001	0.083 ± 0.002	0.077 ± 0.002	0.088 ± 0.002
	15 days	0.097 ± 0.002	0.058 ± 0.002	0.068 ± 0.004	0.075 ± 0.009	0.08 ± 0.002
	20 days	0.085 ± 0.004	0.042 ± 0.002	0.061 ± 0.005	0.069 ± 0.003	0.077 ± 0.001
Chewiness/Mj	1 day	0.8 ± 0.01	0.42 ± 0.03	0.72 ± 0.01	0.54 ± 0.02	0.62 ± 0.04
	5 days	2.05 ± 0.04	0.59 ± 0.04	0.81 ± 0.005	0.99 ± 0.07	1.24 ± 0.13
	10 days	1.72 ± 0.07	0.52 ± 0.11	0.58 ± 0.005	1.15 ± 0.02	1.09 ± 0.03
	15 days	1.51 ± 0.04	0.51 ± 0.02	0.44 ± 0.025	0.90 ± 0.04	0.94 ± 0.09
	20 days	1.35 ± 0.09	0.35 ± 0.02	0.41 ± 0.02	0.79 ± 0.02	0.89 ± 0.09

The texture of the fermented milks was found to be dependent on its composition, particularly the protein and fat concentration (Salvador & Fiszman, 2004). Atamian et al. found that increasing fat levels resulted in decreased hardness in yogurt (Atamian et al., 2014). In this study, the results indicated that the texture could also be influenced by the fermented bacteria. In addition, the protein–polysaccharide interactions and the aggregation and gelation behavior show significant importance in the structure, rheological properties and physical stability of multicomponent food systems (Benbettaieb et al., 2016). The polysaccharide–protein interaction may depend on various environmental conditions, such as pH, temperature and the charge of the biopolymer additive (Ghosh & Bandyopadhyay, 2011). When the solution pH is near the protein isoelectric point (pI), the chance of forming a weak complex from the polysaccharide–protein interaction is greater than when the pH is higher than the protein pI. Hence, combining the variation trend of the texture profile with the pH and acidity, the differences in the texture of the fermented milks in this work might be caused by the different acid‐producing capacities of the fermented bacteria.

### 3.7 Rheological profile of the fermented milks

**FIGURE 4:**
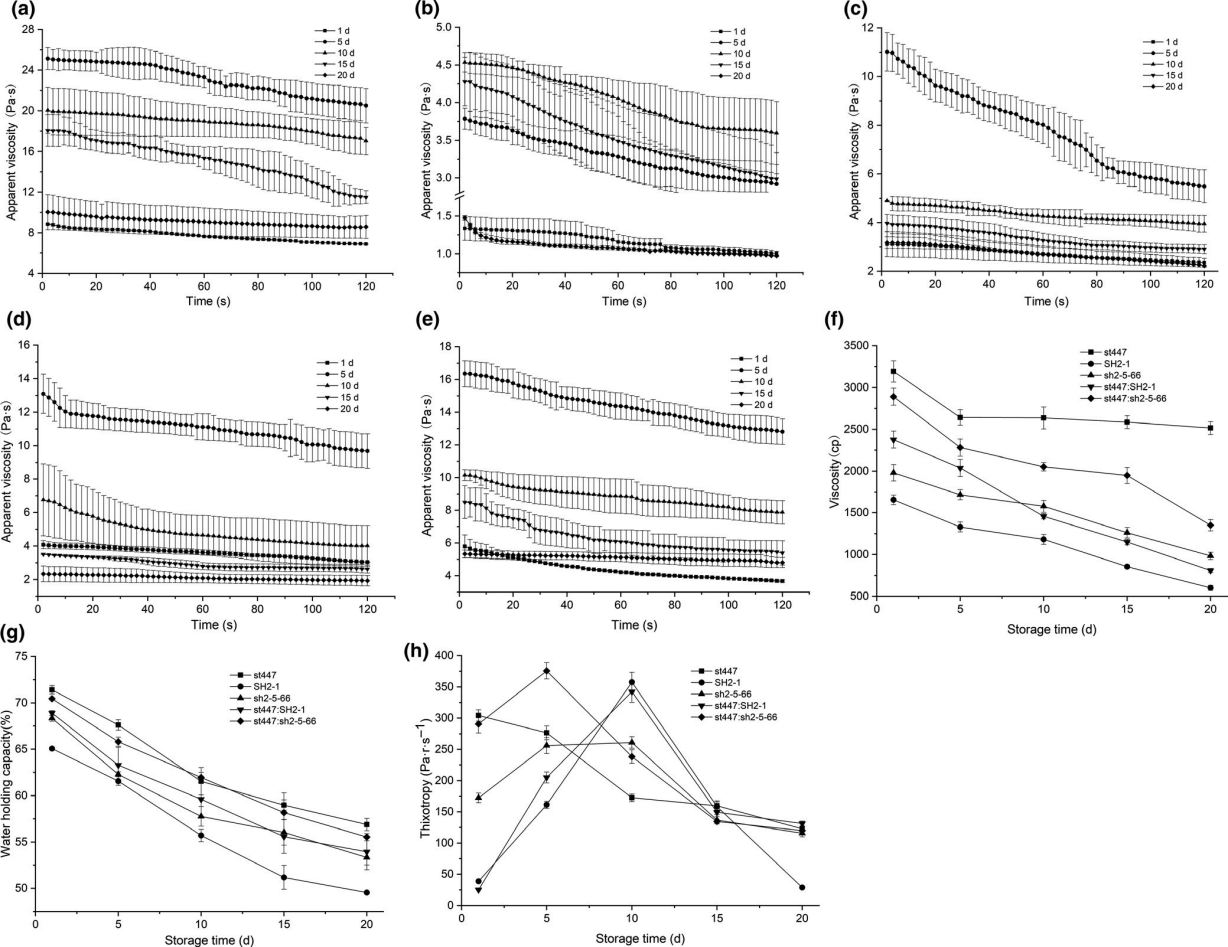
Rheological‐related properties of the fermented milk. Apparent viscosity of milk fermented with st447 (a) SH2‐1, (b) sh2‐5–66, (c) st447 and SH2‐1, (d) st447, and sh2‐5–66 (e), viscosity (f), water content, (g) and thixotropy (h)

The apparent viscosity (Figure 4a–e), viscosity (Figure 4f), water holding capacity (Figure 4g), and thixotropy (Figure 4h) of the samples were estimated. Regarding the period of storage, the milk fermented with sh2‐5–66 showed a higher apparent viscosity than that of SH2‐1, and st447 showed the highest apparent viscosity compared with the other four samples. The apparent viscosity of the fermented milks is associated with the aggregation of casein micelles and gel formation. There are some factors that could influence this process, such as polysaccharide content and pH (Kristo et al., 2011). The apparent viscosity has been reported to increase as the pH of the fermented milk decreases, which is attributable to the additional swelling of casein micelles (Cruz et al., 2013). In this study, the pH continued to decrease during storage, while the apparent viscosity continued to increase before day 5 and then decreased. Therefore, the apparent viscosity could be affected by a specific pH range.

There were significant differences in the viscosities among all the samples ranging from 1656 (SH2‐1) to 3,193 (st447) on day 1 and 600 (SH2‐1) to 2,515 (st447) on day 20. The milk fermented with st447 exhibited the highest viscosity of 3,193 and the lowest variation (678). The viscosities of the sample fermented with sh2‐5–66 were 19.6% and 63.8% higher than that of SH2‐1 on day 1 and day 20, respectively. Moreover, the viscosities of the samples fermented by st447 + sh2‐5–66 and st447 + SH2‐1 were 910 and 720 higher, respectively, than those of samples fermented by sh2‐5–66 and SH2‐1 on day 1.

Water holding capacity is a critical parameter in yogurt manufacturing since it is related to syneresis, which is undesirable. The water holding capacity of all the samples decreased with storage time, ranging from 65.7% (SH2‐1) to 71% (st447) on day 1 and 49.55% (SH2‐1) to 56% (st447) on day 20. The variation in each sample was approximately 15%, and this syneresis was much lower than that of the buffalo yogurt throughout a storage period of 28 days (Nguyen et al., 2014).

Thixotropy was estimated by the hysteresis loop area of the flow curve. All samples showed thixotropic behavior, which was observed in other studies on dairy foods (Paseephol et al., 2008). The thixotropy ranged according to the apparent viscosity during the storage time. The thixotropy of single and cocultured samples with sh2‐5–66 was almost the same as that of st447 but these values were significantly higher than that of SH2‐1 on day 1 and day 20. For single fermentation, the thixotropy of milk fermented with sh2‐5–66 was 3.52 and 2.99 times higher than that of the sample with SH2‐1 on day 1 and day 20, respectively. These data indicated that, compared with SH2‐1, the milks fermented with sh2‐5–66 are less sensitive to shear and less susceptible to breakdown in the network structure.

### 3.8 Microstructures of the fermented milks

**FIGURE 5:**
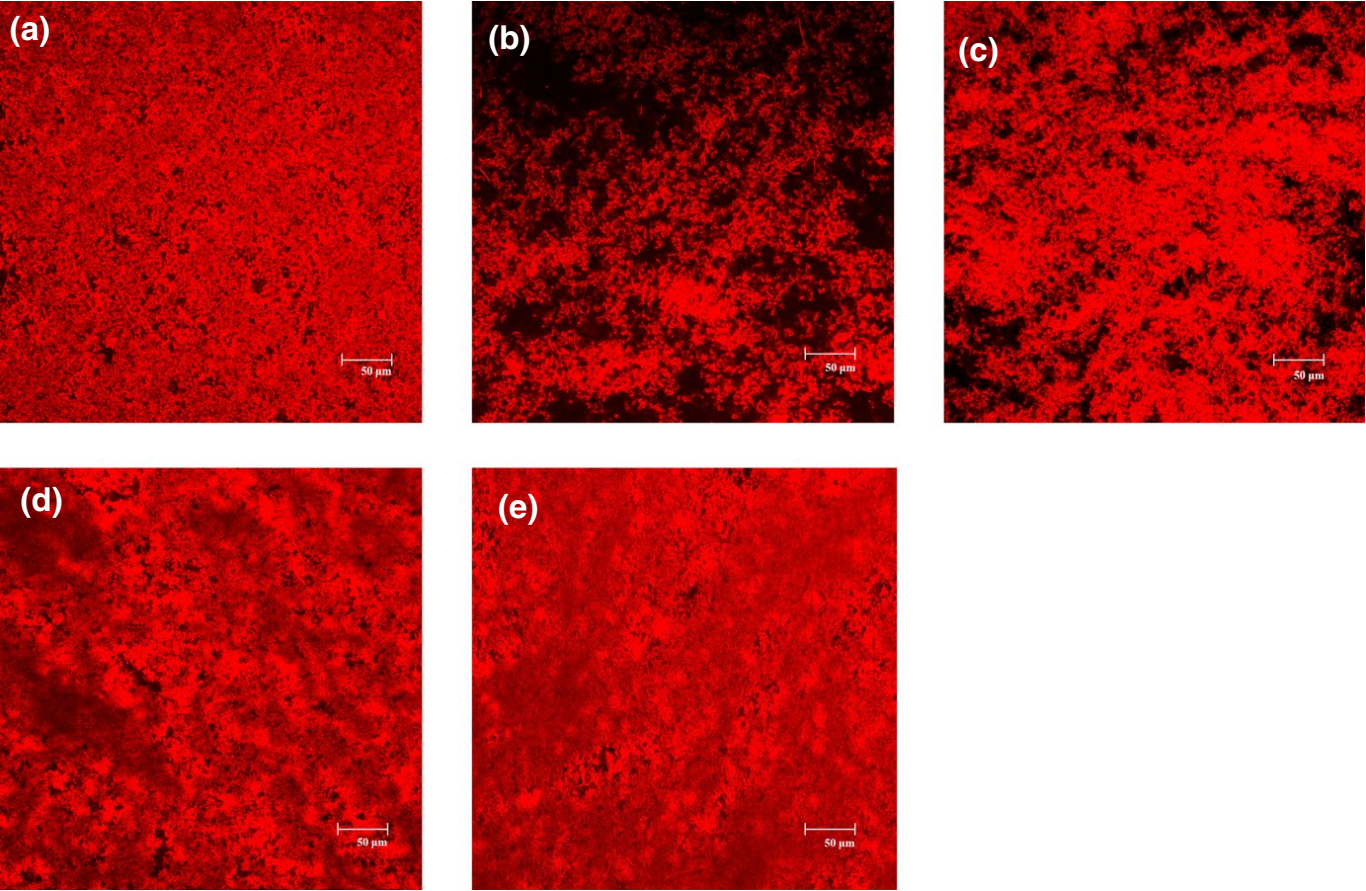
Confocal laser scanning micrographs of milk fermented with st447 (a), SH2‐1 (b), sh2‐5–66 (c), st447 mixed with SH2‐1 (d), and st447 mixed with sh2‐5–66 (e). Bar = 50 μm

The microstructures of the fermented milks were observed by confocal laser scanning microscopy (CLSM). The protein network was stained with rhodamine B and appeared red in the images (Figure 5). The milk fermented with sh2‐5–66 showed a network of thick and continuous protein aggregates with large void spaces, while the milk fermented with SH2‐1 formed a network with many larger pores and channels and less compact protein aggregates (Figure 5b,c). The microstructures of these two samples were different from that of st447, which showed a network of aggregated protein networks with more connectivity between the protein strands and smaller spores (Figure 5a). Moreover, the microstructural properties of milks fermented with sh2‐5–66 or SH2‐1 could be greatly improved by coculture with st447 (Figure 5d,e).

### 3.9 Flavor profile of the fermented milk

The flavor substances in fermented milk are abundant, including aldehydes, ketones, alcohols, esters, and so on (Tian et al., 2017). However, not all of the identified compounds are of sensory importance. Only acetaldehyde, ethanol, acetone, diacetyl, and 2‐butanone have a high impact on the desired production flavor. The major flavor compounds in yogurt used for flavor judgment are acetaldehyde and diacetyl (Erkaya & Sengul, 2011). Acetaldehyde, which possesses a fresh, fruity, and pungent taste, is mainly produced by starter cultures from lactose metabolism as a result of pyruvate decarboxylation or through the formation of the intermediate acetyl coenzyme A. Diacetyl, which contributes buttery, fatty, and pungent notes, contributes to the delicate, full flavor of yogurt and is especially important if the acetaldehyde content is low (Kaminarides et al., 2007).

**FIGURE 6:**
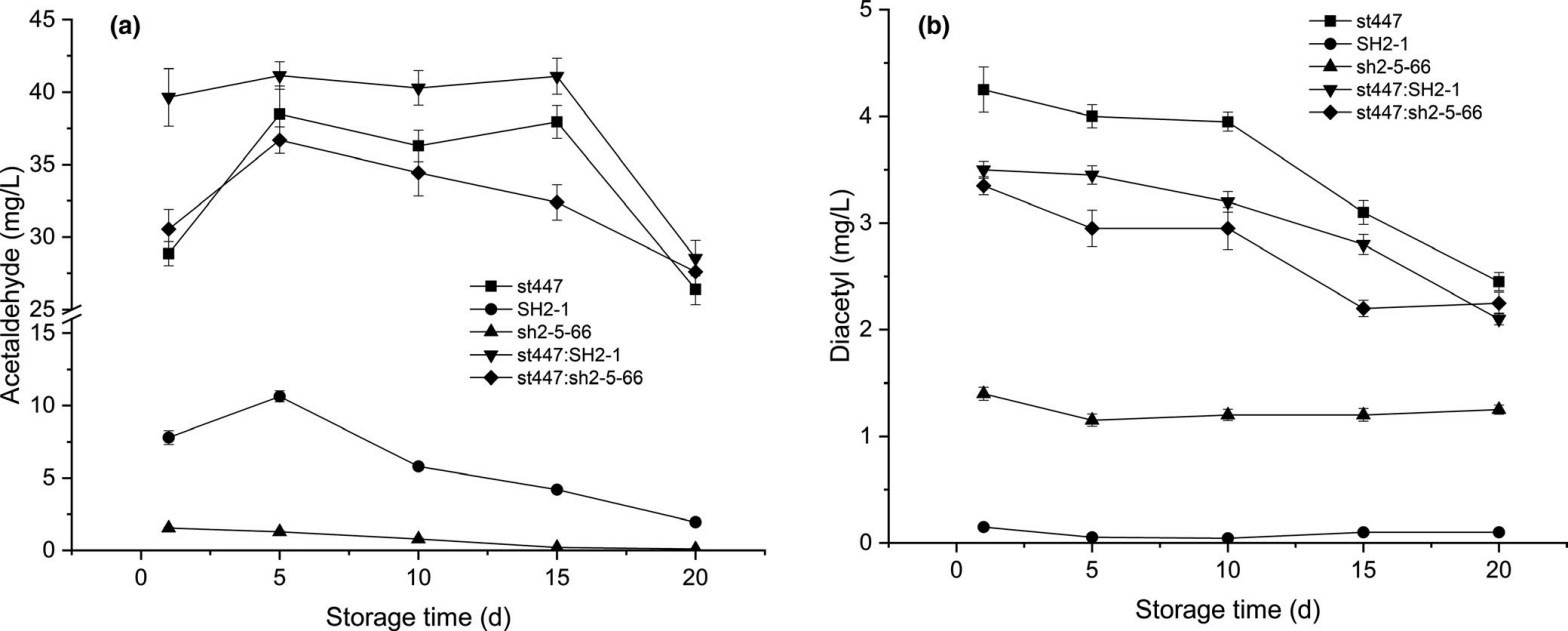
Acetaldehyde (a) and diacetyl (b) content of the fermented milks during storage

In this study, the acetaldehyde and diacetyl contents of the cocultured fermented milks and milk fermented with st447 changed slightly during storage for 15 days and then began to decrease (Figure 6). The acetaldehyde and diacetyl contents of milk fermented with st447 were obviously higher than those of sh2‐5–66 and SH2‐1 during storage. Moreover, during storage, the acetaldehyde and diacetyl contents of the milk fermented with sh2‐5–66 were obviously different with those of SH2‐1, indicating that lactose metabolism might also be affected by UV mutation. Although these two flavor compounds of the milk fermented with sh2‐5–66 were low, the flavor profile could be improved by coculture with st447. In addition, the combination of probiotic culture and substrates such as whey protein, β‐glucan, and fruits could result in satisfactory sensory scores as well as a stronger coagulum and increased firmness and adhesiveness values for the yogurt (Cheng, 2010; Sahan et al., 2008).

## 4 CONCLUSION

In this work, first, the H^+^‐ATPase activity was demonstrated to be highly related to the postacidification of *L. helveticus* SH2‐1. Then, by detecting the H^+^‐ATPase activity, the mutant of *L. helveticus* SH2‐1 named *L. helveticus* sh2‐5–66 with weak postacidification was selected from 80 UV mutants. *Lactobacillus helveticus* sh2‐5–66 exhibited genetic and acid production stability during continuous passage culture for 100 generations. Compared with the milk fermented with the wild‐type strain *L. helveticus* SH2‐1, the milk fermented with *L. helveticus* sh2‐5–66 showed weaker postacidification, better textural and rheological properties and less acetaldehyde and diacetyl during 20 days of storage. Moreover, these main quality characteristics of the milk fermented with *L. helveticus* sh2‐5–66 could be further improved by coculture with the commercial starter *S. thermophilus* st447. These results suggested that *L. helveticus* sh2‐5–66 could be exclusively used for the production of new dairy products.

## CONFLICT OF INTEREST

All the authors have no competing interests.

## Supporting information

App S1

## Data Availability

The data that support the findings of this study are available from the corresponding author upon reasonable request.
