# Supplementary material for: A weak post‐acidification Lactobacillus helveticus UV mutant with improved textural properties
Source: Food Sci Nutr. 2020 Nov 15;9(1):469–79. doi: 10.1002/fsn3.2016 (PMC7802530; doi:10.1002/fsn3.2016)
Supplement: Supplementary file 1 — App S1 [file FSN3-9-469-s001.docx]

**Appendix S1**

**Table S1** UV exposure time and the lethality rate

| **Exposure time (s)** | **Viable counts (CFU/mL)** | **Lethality rate (%)** |
| --- | --- | --- |
| 0 | 1.8*10^9^ | 0 |
| 10 | 1.34*10^9^ | 25.6 |
| 20 | 1.03*10^9^ | 42.7 |
| 30 | 5.67*10^8^ | 68.5 |
| 40 | 4.97*10^8^ | 72.4 |
| 50 | 2.32*10^8^ | 87.1 |
| 60 | 1.26*10^7^ | 99.3 |

**Table S2** H^+^-ATPase activity (U) of the selected colony

| **Colony** | **Enzyme activity** | **Colony** | **Enzyme activity** | **Colony** | **Enzyme activity** | **Colony** | **Enzyme activity** |
| --- | --- | --- | --- | --- | --- | --- | --- |
| 1 | 3.02 | 15 | 3.75 | 29 | 3.34 | 43 | 4.62 |
| 2 | 0.99 | 16 | 3.31 | 30 | 4.27 | 44 | 0.03 |
| 3 | 1.07 | 17 | 4.47 | 31 | 3.40 | 45 | 0.49 |
| 4 | 3.19 | 18 | 2.90 | 32 | 2.64 | 46 | 2.58 |
| 5 | 3.02 | 19 | 3.05 | 33 | 2.64 | 47 | 4.44 |
| 6 | 5.25 | 20 | 1.22 | 34 | 2.70 | 48 | 5.02 |
| 7 | 3.75 | 21 | 1.05 | 35 | 2.70 | 49 | 1.19 |
| 8 | 2.09 | 22 | 3.43 | 36 | 3.43 | 50 | 4.04 |
| 9 | 2.29 | 23 | 2.73 | 37 | 3.34 | 51 | 1.34 |
| 10 | 2.03 | 24 | 2.15 | 38 | 3.92 | 52 | 1.86 |
| 11 | 1.22 | 25 | 2.61 | 39 | 3.48 | 53 | 2.87 |
| 12 | 1.97 | 26 | 2.03 | 40 | 4.33 | 54 | 3.75 |
| 13 | 2.06 | 27 | 2.06 | 41 | 2.03 | 55 | 2.44 |
| 14 | 3.02 | 28 | 1.25 | 42 | 2.12 | C^1^ | 2.32 |

C^1^: *L. helveticus* SH2-1

**Figure S1**





**Figure legend**

Figure S1 Rheological-related properties of the milk fermented by strains SH2-1 and sh2-5 separately mixed with st447 with different blending ratio. Water content (a); Thixotropy (b); Apparent viscosity (c); Viscosity (d).
